# Lesions that do or do not impair digit span: a study of 816 stroke survivors

**DOI:** 10.1093/braincomms/fcab031

**Published:** 2021-03-10

**Authors:** Sharon Geva, Teodros Truneh, Mohamed L Seghier, Thomas M H Hope, Alex P Leff, Jennifer T Crinion, Andrea Gajardo-Vidal, Diego L Lorca-Puls, David W Green, PLORAS Team, Cathy J Price

**Affiliations:** 1 Wellcome Centre for Human Neuroimaging, University College London, London WC1N 3AR, UK; 2 University College London Medical School, London WC1E 6BT, UK; 3 Cognitive Neuroimaging Unit, Emirates College for Advanced Education, Abu Dhabi, UAE; 4 Department of Biomedical Engineering, Khalifa University of Science and Technology, Abu Dhabi, UAE; 5 Institute of Cognitive Neuroscience, University College London, London WC1N 3AR, UK; 6 Department of Brain Repair and Rehabilitation, UCL Queen Square Institute of Neurology, London WC1N 3AR, UK; 7 Department of Speech, Language and Hearing Sciences, Faculty of Health Sciences, Universidad del Desarrollo, Concepcion 4070001, Chile; 8 Department of Experimental Psychology, Faculty of Brain Sciences, University College London, London WC1E 6BT, UK

**Keywords:** aphasia, lesion analysis, verbal short-term memory

## Abstract

Prior studies have reported inconsistency in the lesion sites associated with verbal short-term memory impairments. Here we asked: How many different lesion sites can account for selective impairments in verbal short-term memory that persist over time, and how consistently do these lesion sites impair verbal short-term memory? We assessed verbal short-term memory impairments using a forward digit span task from the Comprehensive Aphasia Test. First, we identified the incidence of digit span impairments in a sample of 816 stroke survivors (541 males/275 females; age at stroke onset 56 ± 13 years; time post-stroke 4.4 ± 5.2 years). Second, we studied the lesion sites in a subgroup of these patients (*n* = 39) with left hemisphere damage and selective digit span impairment—defined as impaired digit span with unimpaired spoken picture naming and spoken word comprehension (tests of speech production and speech perception, respectively). Third, we examined how often these lesion sites were observed in patients who either had no digit span impairments or digit span impairments that co-occurred with difficulties in speech perception and/or production tasks. Digit span impairments were observed in 222/816 patients. Almost all (199/222 = 90%) had left hemisphere damage to five small regions in basal ganglia and/or temporo-parietal areas. Even complete damage to one or more of these five regions was not consistently associated with persistent digit span impairment. However, when the same regions were spared, only 5% (23/455) presented with digit span impairments. These data suggest that verbal short-term memory impairments are most consistently associated with damage to left temporo-parietal and basal ganglia structures. Sparing of these regions very rarely results in persistently poor verbal short-term memory. These findings have clinical implications for predicting recovery of verbal short-term memory after stroke.

## Teaser

Geva et al. identified five lesion sites that caused verbal short-term memory impairments in a sample of 816 stroke survivors. These sites were located in left temporo-parietal and left basal ganglia structures. Sparing of these regions very rarely resulted in persistently poor verbal short-term memory.

## Graphical Abstract

**Graphical Abstract:**
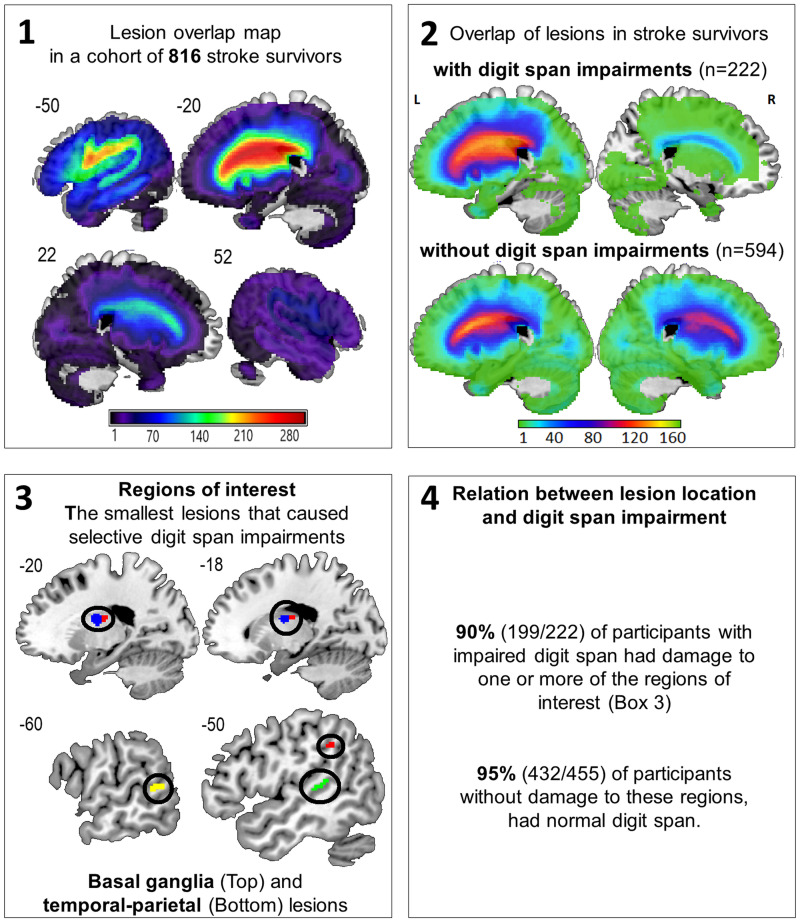

## Introduction

Verbal short-term memory (VSTM) is the passive storage of verbal information. It is limited in its capacity and in the time it can hold information. It requires minimal attentional and other executive resources.^1^^,^^2^ Impaired VSTM is common in patients with aphasia. It affects the ability to immediately recall long sequences of auditory verbal material such as lists of words or digit strings. Many studies suggest that a deficient VSTM can lead to difficulties with language comprehension.^3–6^ Importantly, preliminary work suggests that VSTM impairments are amenable to treatment^7^ which may also facilitate language comprehension.

The goal of our study was to investigate which lesion sites do, and do not, cause persistent VSTM impairments. Such information is essential for predicting which patients will recover from early VSTM impairments and which patients may need sustained therapeutic intervention. Previous lesion studies have associated VSTM impairments with damage to several different brain regions. Early case studies of patients with post-stroke aphasia highlighted the importance of the left inferior parietal lobe (BA40).^3–5^^,^^8–11^ This conclusion was further endorsed by group studies of patients,^12–15^ and a study of direct cortical stimulation.^16^ Other patient studies have associated VSTM with the left posterior superior temporal gyrus,^8^^,^^13^^,^^17–20^ the left inferior frontal gyrus^14^^,^^16^^,^^19^ and the bilateral fronto-polar cortex.^21^

Taken together, it is clear that VSTM impairments can be caused by several different lesion sites but it is unclear: (i) how consistently damage to each of the identified regions impairs VSTM; (ii) whether there are other, as yet unidentified sites, where damage can also impair VSTM; and (iii) which lesion sites are seldom associated with persistent VSTM or cause only transient VSTM impairments. Answering these questions requires large samples of patients who do, and do not, have VSTM impairments. Moreover, we need new lesion identification methods. Current voxel-based lesion-symptom mapping techniques cannot answer the above questions because they: (i) search for the most significant effects across all lesions rather than identifying a set of regions that are each sufficient to cause a deficit, (ii) may miss lesion sites where the effect of damage is inconsistent across patients^22^ and (iii) do not identify lesion sites that are not associated with persistent VSTM impairments. To address our questions of interest, and avoid these caveats, the current study (i) included a large sample of 816 patients, with and without VSTM impairment, (ii) identified all the regions that were damaged in patients with VSTM impairments, (iii) examined how consistently damage to, or preservation of, these sites was observed in patients with normal VSTM and (iv) conducted a linear stepwise regression to test the extent to which the severity of VSTM impairment was explained by lesion site, time post-stroke, age at test and lesion volume.

We follow contemporary models distinguishing (V)STM from working memory, with the latter seen as an active storage requiring controlled and effortful processing.^2^^,^^23–25^ For example, active maintenance of VSTM includes articulatory rehearsal (according to Baddeley and colleagues),^1^^,^^26^ active allocation of attention to the information to be remembered^2^ or a combination of these processes.^27^^,^^28^ While these models agree that short-term memory activates long-term memory traces,^2^^,^^27^^,^^29^ some suggest that both maintenance and refreshing of memory traces require attention,^28^ whereas others argue that maintenance of information in short-term memory can be achieved by off-loading it back to long-term memory, therefore minimizing the demands on attentional resources.^30^ In brief, although any memory task may tap into both the active (working memory) and passive (short term) memory systems, some tasks emphasize one operation over the other.^31^ VSTM is often assessed using simple span tasks which require free recall, without a delay, of words or digits presented in lists of various length.^24^^,^^25^^,^^32^ Here we used the forward digit span task, which is the trademark test of VSTM,^23^^,^^24^ and the most frequently used task in clinical settings today.^33^^,^^34^ Contrary to previous lesion-symptom mapping studies,^12^^,^^13^^,^^17^^,^^19^^,^^20^ here we (i) factored out co-occurring deficits within- rather than between-subjects, by focusing on the lesion sites of patients who had digit span impairments together with normal performance on tests of speech perception and production (i.e. ‘selective digit span impairments’); (ii) minimized the influence of co-occurring damage by identifying the smallest lesions that were sufficient to explain selective digit span impairments in all participants; and (iii) investigated how consistently damage to these regions was observed in other patients with normal digit span, or non-selective digit span impairments. This allowed us to assess inter-subject consistency and inconsistency in the two-way mapping between lesion site and digit span impairments.

## Materials and methods

The study was approved by the London Queen Square Research Ethics Committee. All participants gave written informed consent prior to participation according to the Declaration of Helsinki and were paid £10 per hour compensation for their time.

### Participants

Participants were selected from the Predicting Language Outcome and Recovery After Stroke (PLORAS) database that records behavioural, demographic and imaging data from participants with a history of adult stroke as defined by a neurologist.^35^ At the time of study, our database included 816 patients who: (i) were raised using English as their native language; (ii) were right-handed prior to their stroke; (iii) had no documented or self-reported hearing impairments; and (iv) had structural brain damage reported by a neurologist. For demographics and behaviour of the participants see Table 1. For a lesion overlap map see Supplementary Fig. 1.

### Behavioural assessment

All participants were administered the Comprehensive Aphasia Test,^36^ in a quiet room in our research facility. Selective VSTM impairment was defined as a digit span impairment that occurred in the presence of normal performance on tests of spoken picture naming and spoken word comprehension (tests of speech production and perception that both place minimal demands on VSTM). To further characterize the behavioural profile of the patients defined here as having ‘selective digit span impairment’, we report their performance on semantic and phonological verbal fluency tasks, and word and non-word repetition (see Table 1 and Supplementary section ‘Interaction between digit span impairment, lesion site and other behavioural measurements’ for further methods and results).

#### Forward digit span

Participants heard a list of digits with instructions to repeat the digits immediately in the same order. The task has six progressive levels of difficulty that start with two digits and build up to seven, with a pair of digit strings at each level. Participants had to repeat one string at each level correctly to proceed to the next level. Testing stopped when both strings at one level were incorrect. Phonemic, apraxic and dysarthric errors were not penalized. A digit span of four or below is considered impaired.

#### Spoken picture naming and spoken word comprehension

Scores for spoken picture naming and spoken word comprehension were based on two points per trial for immediate correct responses; one point per trial for correct responses after a self-correction/delay (>5 s)/repetition of stimuli by the examiner; and zero points for trials with incorrect responses.

For spoken picture naming, on each of 24 trials, a line drawing of an object (e.g. knife) was presented, with instructions to name it aloud. Articulatory errors (e.g. dysarthric distortions) not affecting the perceptual identity of the target were scored as correct. Verbal, phonemic, neologistic and dyspraxic errors were scored as incorrect. The maximum score is 48. A score of 43 or below is considered impaired.

For spoken word comprehension, on each of 15 trials, participants heard an object name and pointed to one of four black-and-white line drawings in a 2 × 2 array to indicate which corresponded to the heard word. The three distractor drawings corresponded to object names that were either phonologically or semantically related, or unrelated to the target word (i.e. one of each). The maximum score is 30. A score of 25 or below is considered impaired.

### MRI data acquisition, pre-processing and lesion identification

Four different MRI scanners (Siemens Healthcare, Erlangen, Germany) were used to acquire the structural images, each with 176 sagittal slices, a matrix size of 256 × 224 and a final spatial resolution of 1 mm isotropic voxels. Four hundred and thirty participants were scanned at 3T, with a Trio or Allegra scanner (repetition time/echo time/inversion time =7.92/2.48/910 ms) and 386 were scanned at 1.5 T with a Sonata (repetition time/echo time/inversion time = 12.24/3.56/530 ms) or Avanto (repetition time/echo time/inversion time = 2730/3.57/1000 ms) scanner. An optimized 3D modified driven equilibrium Fourier transform (MDEFT) sequence was used for all scans, except those acquired on the Avanto (*n* = 122), which were reconstructed using a 3D magnetization-prepared rapid acquisition gradient-echo (MPRAGE) sequence.

The T_1_-weighted anatomical whole-brain volume of each participant was analysed with our automated lesion identification toolbox implemented in the Statistical Parametric Mapping software (SPM12; Wellcome Centre for Human Neuroimaging, London, UK; https://www.fil.ion.ucl.ac.uk/spm/, 15 March 2021, date last accessed), running in MATLAB environment (2018a Mathworks, Sherbon, MA, USA).^37^ This converts a scanner-sensitive raw image into a quantitative assessment of structural abnormality that is independent of the scanner used. The procedure combines a modified segmentation-normalization routine with an outlier detection algorithm that identifies whether each voxel is an outlier in relation to normal (control) brains. The output is a binary image that delineates the lesion(s) and here was used to estimate lesion volume, calculate per cent overlap between different lesions, and create lesion overlap maps. All lesions were inspected by eye. The cluster size threshold for lesion identification was initially set at the default (100 contiguous voxels) for all lesions. However, this threshold failed to identify some of the lesions associated with selective digit span impairments. In these cases, we reduced the cluster extent threshold to 20 contiguous voxels and visually inspected the result. For consistency, we employed the same cluster extent threshold (i.e. 20 contiguous voxels) for all participants in the cohorts used to test the variability in the lesion–deficit relationships. In three participants with a selective digit span impairment (one with left hemisphere damage and two with bilateral damage), the lesion was incompletely identified by the automated lesion detection algorithm, even when the cluster extent threshold was 20 contiguous voxels. In these cases, we (i) illustrate the lesions by manually outlining the lesioned region on MRI images and (ii) verified how frequently damage to the identified regions was associated with digit span impairments using the PLORAS ‘neurological description’ records.

### Lesion identification method

We previously reported a voxel-based lesion-symptom mapping study of digit span/VSTM in 210 patients,^17^ most of them included in our current sample. This analysis was repeated with our larger sample. The results show a highly significant relationship between digit span and brain structure in a very extensive left perisylvian region (see Supplementary section ‘Identifying regions associated with digit span impairment using Voxel-based Morphometry’ and Supplementary Fig. 2). This method does not allow us to distinguish the multiple different lesion sites within this area that are sufficient to impair digit span. We therefore adopted the following analysis: Lesions were identified iteratively within the group of participants with selective VSTM impairments. Our goal was to identify the smallest regions where damage was sufficient to account for the maximum number of participants with selective VSTM impairments. The lesion image of the participant with the smallest lesion acted as the first region of interest (ROI). We calculated the degree of damage to this ROI for all other participants, by quantifying the percentage of overlap between each participant’s binary lesion image and the ROI (varying between 0% and 100%). The second ROI was the smallest lesion that did not substantially damage the first ROI (<50% overlap). The process was repeated until all participants were defined as having ≥50% damage to one of the ROIs. The ROIs were then grouped by anatomical criteria. Such grouping is motivated because some of the ROIs were very close to one another and therefore commonly damaged together, making it difficult to dissociate their unique effect on behavioural performance.

### Analysis steps

We divided the sample into three cohorts: participants with left hemisphere (LH) lesions, right hemisphere (RH) lesions or bilateral lesions. Each cohort included participants with: selective digit span impairments, non-selective digit span impairments or normal digit span.

#### Digit span impairments after unilateral damage

In Step 1, we identified lesion sites associated with selective digit span impairments (see ‘Lesion identification method’). In Step 2, we tested how well the presence or absence of digit span impairments in all other participants with LH or RH damage was explained by the degree of damage to the ROIs identified in Step 1.

#### Digit span impairments after bilateral damage

In the case of bilateral damage, we initially determined whether participants with digit span impairments had damage to any of the LH or RH ROIs identified above and then analysed the lesion sites of participants with selective digit span impairments but with no damage to the LH or RH ROIs identified above.

#### Inter-participant variability in the effect of lesions on digit span ability

We investigated inter-participant variability in the effect of damage on digit span ability among all the participants with lesions affecting the left hemisphere (*n* = 473 with left hemisphere lesions, *n* = 154 with bilateral lesions). In this cohort of 627 patients, we studied the influence of: (i) the degree of ROI damage and co-occurring damage to multiple ROIs and (ii) the additional effect of time between stroke and testing, age at testing and lesion volume.

- The degree of ROI damage and co-occurring damage to multiple ROIs: The influence of the degree of ROI damage was tested by subdividing participants into 12 groups, according to the degree of damage to each ROI, ranging from no damage (0%) to complete damage (100%), and 10 levels of partial damage: 1–9%, 10–19%, 20–29%, 30–39%, 40–49%, 50–59%, 60–69%, 70–79%; 80–89%, 90–99%. When the degree of damage to additional ROIs is taken into account, the number of groups escalates (e.g. with two ROIs we would have 12 × 12 = 144 groups). We therefore reduced the number of intermediate groups by collapsing over those with (i) few or no participants, or (ii) with the same proportion of participants with digit span impairments. For example, the incidence of digit span impairments was very low for participants with 1–9% or 10–19% damage to all ROIs, we therefore collapsed over these two groups. The number of groups was also reduced by pooling participants who had damage to the same anatomical area. For example, there were two ROIs in the left basal ganglia (BG ROI-1 and BG ROI-2) that were very close to one another and typically damaged together. Participant assignment was based on the maximum degree of damage to any one of the ROIs within an anatomical area (i.e. participants with 100% damage to BG ROI-1 were grouped with participants with 100% damage to BG ROI-2). This avoided empty cells and increased the number of participants in each group. We ended up with three levels of damage to two anatomical areas which together constituted 3 × 3 = 9 groups. Group differences in the incidence of digit span impairments were tested using chi-square tests with *P-*values Bonferroni corrected for multiple comparisons.
- Controlling for time post-stroke, age at testing and lesion volume: we tested whether damage to our ROIs explains variance in digit span ability, over and above variability explained by time post-stroke, age at testing and lesion volume. To this end, we used a linear regression, entering time post-stroke, age at testing and lesion volume as predictive variables in Step 1, and degree of damage to our ROIs in Step 2. Degree of damage was categorized at three levels, as described above. All assumptions for multiple regression were met in our data, and only one of 627 cases was classified as an outlier according to Cook’s distance value, at *P* < 0.05.

### Data availability statement

The data that support the findings of this study are available from the senior author, upon reasonable request.

## Results

### Digit span impairments after left hemisphere damage

Of the 473 patients with left hemisphere damage, 39 had selective digit span impairments, 152 had non-selective digit span impairments and 282 had normal digit span. These three groups did not significantly differ in time post-stroke (Mann–Whitney *U* Test, *P* ≥ 0.10 for all comparisons). As expected, patients with preserved digit span had the smallest lesions, whereas those with non-selective digit span impairment had the largest lesions (Mann–Whitney *U* Test, *P* < 0.01 for all comparisons).

Step 1: Focusing on the 39 patients with selective digit span impairments (see Table 1 for demographics and behavioural scores), we identified five small left hemisphere regions of interest which were damaged in more than one participant with impaired digit span. Three lesions were in temporo-parietal (TP) areas: (i) cortex either side of the superior temporal sulcus—BA 22 (Fig. 1, green); (ii) planum temporale—BA 42 (Fig. 1, cyan); (iii) lateral middle temporal gyrus—BA 21 and left parietal cortex—BA 40 (Fig. 1, blue); and two lesions were in the BG: (iv) body of caudate nucleus and posterior putamen (Fig. 1, yellow), (v) posterior putamen (Fig. 1, red). Damage to one or more of these five ROIs was observed in 90% (35/39 participants) of this sample. The lesion sites in the other four participants were non-overlapping with each other or with the first five ROIs (Fig. 2).

Step 2: Focusing on the remaining 434 participants with left hemisphere damage (152 with non-selective digit span impairments and 282 with normal digit span), we found that when the first five ROIs from Step 1 were all <20% damaged, the incidence of digit span impairments was very low (9/160 = 6%). However, the presence of ROI damage was not consistently associated with impaired digit span, even when the ROIs were 100% damaged (see Table 2 and below for analysis of inter-participant variability in the effect of ROI damage on digit span performance).

### Digit span impairments after right hemisphere damage

Of the 189 participants with RH damage, 97% (183) had normal digit span. The other 3% (six participants) had selective digit span impairments.

Step 1: We identified four regions, one or more of which was damaged in all six participants with selective digit span impairment after RH damage. The four lesions affected: the frontal and premotor cortices (ROI-A, Fig. 3A, green); frontal cortex, insula and BG (ROI-B, Fig. 3A, yellow); thalamus and frontal white matter (ROI-C, Fig. 3A, red); and inferior parietal lobule (ROI-D, Fig. 3a, blue).

Step 2: In the 183 participants with RH lesions and no digit span impairments, 28 had damage to ROI-A and/or ROI-B [i.e. the incidence of impaired digit span among all the participants with RH damage was very low (2/30 = 7%) after damage to ROI-A and/or ROI-B]. Isolated damage to ROI-C or ROI-D was rarely seen in our sample. Only four participants had isolated damage to ROI-C. Therefore, together with the patient defining the ROI, 1/5 had impaired digit span after damage to ROI-C. Likewise, only one patient had isolated damage to ROI-D, therefore, together with the patient defining the ROI, 1/2 had impaired digit span after damage to ROI-D. In the remaining 152 patients with RH damage and normal digit span, 55 had co-occurring damage to at least two of the four RH ROIs (A–D), and 97 spared all four RH ROIs.

### Digit span impairments after bilateral damage

Of the 154 participants with bilateral damage, 25 had digit span impairments (5 selective, 20 non-selective) and 129 had normal digit span. All but four of the 25 (i.e. 21/25 = 84%) with digit span impairments had ≥20% damage to at least one of the first five ROIs identified from patients with unilateral left hemisphere lesions (Table 2). The lesion sites in the four participants (two with selective and two with non-selective digit span impairments) who spared all five left hemisphere ROIs, had <20% overlap with each other and with all of the additional lesion sites identified in either right or left hemispheric participants (Fig. 3B).

### Inter-participant variability in the effect of lesions on digit span ability

#### The degree of ROI damage and co-occurring damage to multiple ROIs

Participants were assigned to different groups according to the degree of damage to the TP and BG ROIs (see Table 3). High damage was categorized when one of the ROIs in an anatomical region (TP or BG) was 90–100% damaged; low damage was categorized when all regions in an anatomical region were <20% damaged; medium damage was the intermediate range (20–89% damage). We found that the incidence of digit span impairments was significantly higher (chi-square test, *P* < 0.05 Bonferroni corrected) for:

- TP damage compared to BG damage: 72% had impaired digit span when TP damage was high and BG damage was low, whereas 24% had impaired digit span when BG damage was high and TP damage was low.
- Increasing degrees of TP damage: 72–81%, 20–62% and 6–27% were impaired when TP damage was high, medium or low (with variance within group depending on the degree of damage to BG ROIs).
- High or medium versus low damage to BG, (24%, 27% and 6% impaired, respectively), when TP damage was low.

#### Controlling for time post-stroke, age at testing and lesion volume

Time post-stroke, age at testing and lesion volume together explained 21.7% of the variance in digit span scores (*F*(3,623) = 57.5, *P* < 0.001). In Step 2, the degree of damage to TP and BG together significantly increased the amount of variance explained by 21.7% (*F*change(2,621) = 119.1, *P* < 0.001), with both variables being significant predictors (*P* < 0.001 for both). In this combined model, the significant predictors were damage to BG (*P* < 0.001), damage to TP (*P* < 0.001) and time since stroke (*P* = 0.002), with lesion volume only showing a trend (*P* = 0.057), and age at testing not significant (*P* = 0.329). This indicates that less damage to our ROIs and longer time since stroke predict higher digit span ability. Results remained similar when excluding the 39 patients who were used to define the ROIs: The degree of damage to our ROIs significantly increased the amount of explained variability in digit span, over and above time post-stroke, age at testing and lesion volume together (*P* < 0.001).

### Summary

Our sample of 816 participants included 222 with digit span impairments: 90% of these (199/222) had 20% or more damage to at least one of five small left TP and BG ROIs. When these small ROIs were all <20% damaged, the incidence of digit span impairments was very low after both left hemisphere (LH) damage (17/266 = 6%, Fig. 4) and RH damage (6/189 = 3%).

## Discussion

Findings from previous studies have been inconsistent in the lesion sites associated with VSTM impairments. Here we sought to characterize the full set of lesion sites that are, and are not, associated with persistently impaired VSTM in a large sample of 816 stroke survivors. This cannot be achieved with current voxel-based lesion-symptom mapping techniques. Our 816 participants included 222 participants who had poor digit span (a sensitive measure of VSTM). A subset of these was used to identify ROIs where damage resulted in ‘selective’ digit span impairments.

We then tested how often damage to these ROIs was observed in all other participants. This revealed two surprising findings. First, 90% (199/222) of participants with impaired digit span had damage in just two anatomical regions (TP regions or BG). Second, when these regions were preserved, 95% (432/455) of participants had normal digit span. These findings have important implications for understanding recovery of VSTM. Specifically, they lead us to hypothesize that participants with impaired VSTM after stroke might be more likely to recover if they have preserved the left BG and left TP ROIs that we have identified here. We plan to test this hypothesis in future studies.

Our results did not lead to the hypothesis that VSTM would be consistently and persistently impaired following damage to any of our ROIs in isolation or in any combination. To the contrary, our results show that many participants with damage to these regions had normal digit span by the time we tested them. As some of the variation in digit span ability was explained by time post-stroke, we infer that slowly evolving functional reorganization and compensatory mechanisms may have supported the recovery of VSTM. Further longitudinal data are required to confirm this by monitoring how VSTM impairment recovers with time, and how this depends on lesion site.

Below we consider what is known about our five ROIs from prior literature, focusing on (i) the temporal and parietal regions that have previously been associated with VSTM, (ii) the reason why BG lesions may also impair digit span, (iii) why we found no evidence that VSTM impairment can be attributed to focal left frontal lesions, (iv) digit span impairments that occurred in patients who had spared our five ROIs and (v) study caveats.

### Left temporal and parietal regions

Our three temporal and parietal ROIs have been associated with digit span impairments in prior studies. Our smallest temporal lobe ROI (Fig. 1, green) was reported in Leff et al.^17^ and is very similar in size and location to the lesion described by Takayama et al.^18^ who, to the best of our knowledge, presented the only other case study of a participant with a small and focal temporal lesion accompanied by a selective digit span impairment. Our planum temporale ROI (Fig. 1, cyan) corresponds to the part of the left temporal lobe most significantly associated with digit span impairments in the group studies by Baldo et al.^13^ and Ghaleh et al.^20^ The lesion affecting the parietal lobe (Fig. 1, blue) encompassed the TP white matter underlying the supramarginal gyrus, in line with many previous findings,^3–5^^,^^10–13^ as well as a temporal region implicated in previous group studies.^13^ It is interesting to note that TMS to a supramarginal site overlapping with our ROI affected immediate recall of a short list of non-words, but not of words^38^ and direct electrical stimulation of the same site in awake patients caused order errors in digit span but not item errors.^16^ These findings suggest that our parietal ROI is required for tasks with low semantic content (like digit span) and that other neural systems might support short term maintenance of semantic content.

We make three novel contributions to our understanding of the relationship between left TP damage and digit span impairments. First, by focusing on small lesion sites, we are able to say which parts of the temporal and parietal lobes are most likely to be critical for VSTM. In contrast, previous group studies have not demonstrated that focal damage to the regions showing a significant relationship between lesion and deficit would be sufficient to cause VSTM impairments. Second, we found that damage to our TP ROIs was frequently associated with digit span impairments and this was not influenced by the degree of co-occurring damage to the BG. Third, by including large cohorts of participants, with and without digit span impairments, we demonstrate that even 100% damage to TP ROIs does not consistently cause persistent digit span impairments. Further studies are required to compare the effect of damage to different parts of the anatomical areas we have identified here because in our sample, damage to one ROI tended to co-occur with damage to other parts of the same anatomical area.

Finally, we emphasize that the temporal and parietal regions identified here are also consistently implicated in studies of language processing, supporting some current views that short-term memory does not rely on distinct neural mechanisms, at least in the spatial resolution allowed by MRI. Rather, regions responsible for processing representations^39–41^ or maintaining them in long-term memory^30^ could also be responsible for maintaining this information short term. A support for this view comes from a recent fMRI study^42^ that associated the encoding of words and non-words lists with activation in the superior temporal gyrus (middle to posterior portion), overlapping with the regions found here. Additionally, the same study showed that activation patterns distinguished words and non-words, suggesting that long-term linguistic knowledge stored in temporal cortex is involved in encoding lists of verbal information.

### Left basal ganglia regions

While BG damage was less consistently associated with digit span impairments than TP damage, we found that some participants with small and focal lesions to the BG had acquired digit span impairments. A meta-analysis of activation studies found that the left BG showed preference to verbal material during working memory storage tasks (defined as simple storage which requires manipulation). The authors suggested that the BG codes serial order.^43^ However, our finding was still unexpected because a prior (smaller) lesion study found no significant evidence of VSTM impairments after subcortical lesions.^44^ Here we can explain the previous null result in terms of inconsistency in the effect of BG damage on VSTM. Indeed, although we demonstrate that some of our participants had selective digit span impairments after damage to specific BG regions, the majority (>75%) with damage to our BG ROIs had normal digit span. Additionally, it might be that, following damage to the BG, compensatory mechanisms can be established relatively quickly. Diao et al.^44^ presented data suggesting that such mechanisms exist in the contra-lesional frontal lobe.

### Lack of evidence associating left frontal lesions with VSTM impairment

A number of previous studies have suggested that the left inferior frontal cortex supports VSTM.^16^^,^^19^ However, we found no evidence that selective VSTM impairments (as defined here) can be attributed to focal lesions to left inferior frontal regions as our database does not include any patients with focal left frontal lobe damage and selective digit span impairments. We therefore conclude that, in our large cohort of patients, selective impairments in a standard forward digit span task are not associated with focal lesions to the left inferior frontal cortex. This conclusion is in line with cumulative evidence suggesting a functional distinction between left temporal/parietal regions supporting storage; and left motor/inferior frontal regions supporting articulatory rehearsal processes.^13^^,^^20^^,^^45^^,^^46^

Dorsal prefrontal involvement in VSTM tasks is more controversial. Some suggest that even simple span tasks require attention allocation for retrieval from long-term memory and for choosing operations according to task demands. This type of attention has been associated with activation in the prefrontal cortex (see review of fMRI studies).^28^ However, the reviewed fMRI studies all used complex working memory tasks, rather than digit span; and a study directly assessing the role of frontal regions showed that superior frontal and orbitofrontal activation was only evident at higher memory loads,^47^ potentially exceeding the capacity of VSTM.^48^ More recently it has been suggested that the role of the prefrontal cortex is to re-activate disrupted memory traces in posterior (sensory) regions, or act as a relay station for higher cognitive processing.^41^ Crucially, these higher-level processes are not associated with the performance of the forward digit span task as we elaborate below.^49^

### Digit span impairments in patients who spared our five ROIs

In addition to identifying the lesion sites that are most consistently observed in patients with digit span impairments, we also identified 23 patients who had digit span impairment, but their lesions did not affect the five ROIs identified in the TP and BG regions.

In 11 of these patients, the digit span impairment was non-selective, the majority of these (*n* = 8) had damage to left frontal and/or insula regions with impaired speech perception and/or production. We cannot therefore rule out the possibility that impaired digit span in these 11 patients was due to concomitant language deficits rather than poor VSTM *per se*.

In the other 12 patients, the digit span impairment was consistent with our definition of selective. Four of these had left hemisphere lesions, 6 had RH lesions and 2 had bilateral damage. Future studies are required to determine whether these lesion sites are: (i) critical for VSTM in the general population, but are infrequently damaged in stroke patients; (ii) frequently damaged but rarely associated with VSTM impairment, perhaps because other regions are available to compensate for their role in VSTM; or (iii) a combination of these factors. For example, 5 of the 12 patients with selective digit span impairment had damage to multiple disparate regions. The exact combination of regions that are damaged is likely to be rare if damage is caused by different ischaemic events. Multiple lesion sites are also more likely to damage the compensatory neural systems that support VSTM recovery. It is also possible that digit span impairment was the consequence of using atypical premorbid cognitive strategies to perform the digit span task. For example, one of the atypical lesions associated with impaired digit span was in the occipital lobe. As occipital areas are often implicated in visual imagery^50^^,^^51^ and some individuals may rely on visual imagery for verbal memory,^52^ its plausible that impaired digit span after the occipital lesion might reflect poor visual imagery in a patient who previously engaged in visual imagery strategies.

Future studies are needed to (i) test this hypothesis; (ii) find new patients with focal lesions to regions that are rarely damaged after stroke and test whether these patients have long-term digit span impairments; (iii) investigate non-lesion factors and inter-patient variability to explain why damage to a region only rarely impairs digit span; and (iv) identify other lesion sites that may be associated with digit span impairments.

### Study caveats

Despite reporting the largest cohort of stroke survivors with digit span impairments ever studied, lesion-deficit conclusions are always limited to the brain regions lesioned in the participants being investigated. The definition of critical lesion sites is also biased towards regions with greater ischaemic vulnerability.^53^ Therefore, there may be regions that are crucial for VSTM but not typically affected by stroke. For example, VSTM impairments have been reported following traumatic brain injury affecting the bilateral fronto-polar cortex^21^ and rTMS to the anterior temporal lobe.^38^ However, such lesions are unlikely to occur as a result of stroke.

There are limitations in assessing VSTM with digit span. Some patients with aphasia have difficulties with numerical skills.^54^ However, in our entire cohort only 24 patients had impaired numerical skills as assessed using the arithmetic subtest in the Comprehensive Aphasia Test, and only half of them had co-occurring digit span impairment, suggesting that in our sample, the two were not highly associated. Second, the use of a single task limits the ability to generalize the results. However, the digit span task we used is the most frequently used clinical assessment of VSTM, and has excellent construct validity and good predictive validity, discriminant validity and test–retest reliability. The task content and concurrent validity are similar to all other VSTM tasks used clinically, except for the digit span task of the Wechsler Adult Intelligence Scale,^33^ which, importantly, is not standardized for patients with aphasia. Indeed, in their comprehensive review, Salis et al.^34^ listed the Comprehensive Aphasia Test digit span task as one of the preferred tests for assessing VSTM in aphasia. Still, future studies should use a battery of VSTM tests^40^ or additional performance metrics.^55–57^ Additionally, while the digit span task used here places minimal demands on attentional mechanisms, future studies should directly evaluate the attention abilities of the patients. We note though that a recent study has shown that while patients with aphasia do exhibit attention impairments, performance on attention tasks did not correlate with performance on the forward digit span task.^49^

Another related limitation is the use of only one test for each cognitive process of interest (word comprehension and production) when defining the selectivity of the digit span impairment. Patients showing normal performance on the picture naming and picture matching tasks may nevertheless present with more subtle phonological impairment which is often associated with damage in the area of the TP ROIs identified here. However, Majerus et al.^57^ have shown that phonological abilities correlated only with some types of VSTM impairments (item errors but not order errors), and that this correlation was not applicable to all study participants. Hence, while some of our patients might have had subtle difficulties with word production and comprehension underlying their digit span impairments, it is reasonable to assume that others did not. In addition, we further investigated whether lesion sites in our group of patients with selective digit span impairments are consistently associated with performance on other related tasks, including semantic and phonological fluency, and word and non-word repetition. We found that there was no interaction between lesion site or degree of damage, and performance on any of these tasks.

Future studies with much larger cohorts of patients are needed to assess how the severity of VSTM impairment is influenced by lesion site and degree of damage. Specifically, we would need to study groups of patients with focal damage to each of our identified lesion sites, and these groups would need to be matched for time post-stroke and co-occurring behavioural impairments. This will require many more patients with VSTM impairments so adequate sample size can be obtained within each lesion site group.

## Conclusions

By analysing behavioural and lesion data from a large cohort of patients, we have shown that 90% of patients with digit span impairments have damage to specific sites in left TP and BG regions. We hypothesize that, if participants with VSTM impairments in the early days of their stroke have preserved all our ROIs, their VSTM is likely to recover. Our findings therefore have potential clinical implications for predicting and explaining recovery of VSTM after stroke as well as contributing to our understanding of the functional anatomy of VSTM.

## Supplementary material

Supplementary material is available at *Brain Communications* online.

## Supplementary Material

fcab031_Supplementary_Data

## Appendix

PLORAS Team members contributed to the acquisition and analysis of behavioural data. They include: Louise Lim, Rachel Bruce, Hayley Woodgate, Sophie Roberts, Kate Ledingham, Shamima Khan, Megan Docksey and Storm Anderson.

## Abbreviations

BG =: basal ganglia
RH =: right hemisphere
ROI =: region of interest
TP =: temporo-parietal
VSTM =: verbal short-term memory
