# Supplementary material for: Lesions that do or do not impair digit span: a study of 816 stroke survivors
Source: Brain Commun. 2021 Mar 10;3(2):fcab031. doi: 10.1093/braincomms/fcab031 (PMC8066865; doi:10.1093/braincomms/fcab031)
Supplement: fcab031_Supplementary_Data [file fcab031_supplementary_data.pdf]

Supplementary Material

Participants

Figure 1. Lesion overlap map of all participants

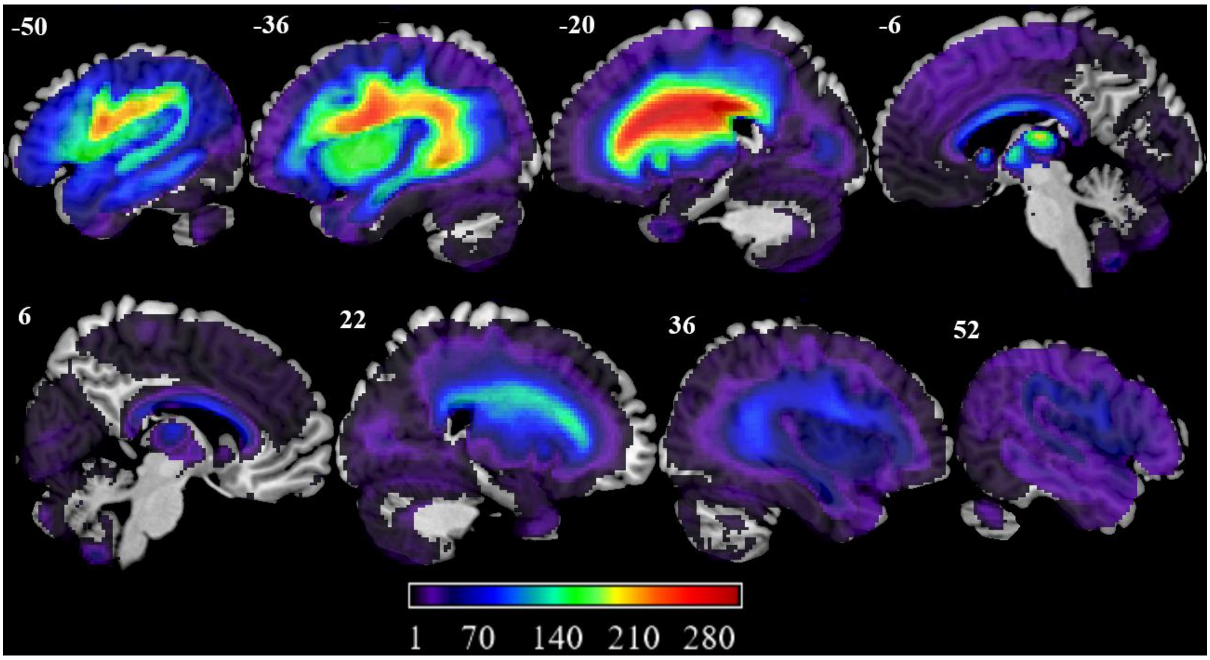

Lesion overlap map of all participants (n=816), shown on sagittal slices in MNI space in the left hemisphere (top) and right hemisphere (bottom). x values are indicated above each slice. The colour scale indicates the number of patients with overlapping lesions at each given voxel.

Interaction between digit span impairment, lesion site and other behavioural measurements

Methods

We assessed whether digit span performance was related to repetition, semantic and phonological abilities, using word and non-word auditory repetition, and semantic and phonological fluency tasks.

Auditory repetition

Scores for auditory repetition were based on two points per trial for immediate correct responses; one point per trial for correct responses after a self-correction / delay (>5s) / repetition of stimuli by the examiner; and zero points for trials with incorrect responses.

Participants were asked to repeat 16 heard words (one or three syllables) and five heard non-words (one or two syllables). Verbal, phonemic, neologistic and dyspraxic errors were marked as incorrect. Dysarthric distortions were marked as correct provided it was clear that each phoneme within the word had been correctly selected.

For auditory word repetition, the maximum T-score is 65, and a T-score of 56 or below is considered impaired. In this test, auditory input and speech production processes of a single word are engaged similarly to the digit span task; the main difference is that for single word repetition, unlike digit span, the capacity of auditory short-term memory is not stretched.

For auditory non-word repetition, the maximum T-score is 67 and a T-score of 52 or below is considered impaired. Unlike word repetition, repetition of non-words cannot be facilitated by word recognition or semantic processing; it is entirely reliant on phonological processing. The memory load may therefore be higher than that required for auditory word repetition but it is lower than that required for digit span.

Verbal fluency

Participants were given 60 s to say as many words according to a semantic prompt (“Name as many animals as you can”) followed by a phonological prompt (“Name words beginning with the letter ‘s’”). Participants were allowed to make articulatory errors but repeated items (perseverations) were not counted. We provide raw scores for the semantic and phonological fluency tests, as well as a combined T-score for the sum of these two component tests. The maximum combined T-score is 75. A T-score of 57 or below is considered impaired. This task has no auditory perceptual component (other than self-monitoring). It is designed primarily to test word retrieval and is commonly used as a test of executive functions. We included this

measure to tap executive demands, sequencing and phonological abilities, all of which are necessary for digit span.

Statistical analysis

To test for the interaction between lesion site and repetition, phonology and semantics, we used two separate MANOVAs. Each MANOVA had two between-subject factors: degree of damage to TP ROIs (three levels: high, medium, low), and degree of damage to BG ROIs (three levels: high, medium, low); and one within subject factor (two levels for 'Repetition': word / non-word in MANOVA-1, two levels for 'Fluency': phonological / semantic in MANOVA-2). Post-hoc tests were performed using independent sample t-tests.

Results

As expected, performance on the fluency tasks was lower among patients with higher degree of damage to TP ROIs (Main effect of damage to TP ROIs, $F = 3.4$, $p = 0.046$). Post hoc independent sample t-tests showed that patients with low degree of damage performed better on both tasks than patients with medium degree of damage ($t = 2.2$, $p = 0.044$, $t = 4.9$, $p < 0.001$ for phonological and semantic fluency, respectively), and on one task compared with patients with high degree of damage ($t = 2.8$, $p = 0.008$ for the semantic fluency task, $p > 0.05$ for the phonological fluency task). There was no evidence to suggest that patients with different degrees of damage to our ROIs systematically differed in their repetition abilities, because the interaction between task performance and lesion group did not reach significance for either of the repetition tasks ($p > 0.05$ for all).

Identifying regions associated with digit span impairment using Voxel-based Morphometry

Method

We used voxel-based morphometry (VBM),[1] performed in SPM12 using the general linear model in order to assess lesion-deficit relationships.[2] The imaging data entered into the analyses were fuzzy lesion images that are produced by our automated lesion identification toolbox. Fuzzy images encode the degree of abnormality on a continuous scale from 0 (completely normal) to 1 (completely abnormal) at each given voxel relative to normative data drawn from a sample of 64 neurologically-normal controls. We choose fuzzy images over segmented grey or white matter probability images which are used in standard VBM routines, as each of the latter by itself do not provide a complete account of the lesion.[3]

The multiple regression model included fuzzy images of all 816 participants and the digit span scores as the regressor of interest. In Model 1 we also included regressors of no interest to factor out variance related to speech production (spoken picture naming), speech comprehension (spoken word comprehension), along with lesion volume, age at test and time since stroke. In Model 2 we aimed to replicate our previous findings from an analysis of 210 patients,[4] by including the following regressors of no interest: word repetition, non-word repetition, verbal fluency, and spoken picture naming, along with lesion volume, age at test, age at test squared, and time since stroke. This model included 813 of 816 patients for whom all scores were available.

The search volume was restricted to voxels that were damaged in at least five patients (see rationale in Sperber et al.[6], and an example in Fridriksson et al.[5]). To this end, a lesion overlap map based on the binary lesion images from all 816 patients was created, thresholded at five, and used as an inclusive mask before estimating the model. Statistical voxel-level threshold was set at $p < 0.05$ after FWE-correction for multiple comparisons (using random field theory as implemented in SPM)[7] across the whole search volume.

Results

Model 1: Digit span was associated with tissue integrity in a very large perisylvian cluster in the left hemisphere (peak coordinate x = -30, y = -36, z = 18, Z-score = inf, cluster size = 14,244 voxels), which included the posterior part of the inferior and middle frontal gyri, putamen, insula, inferior parietal lobe, supramarginal gyrus, middle and posterior superior temporal gyrus, superior temporal sulcus, middle temporal gyrus, as well as parts of the

superior longitudinal fasciculus and corona radiata. Importantly, all five ROIs associated with digit span impairment overlapped with the cluster identified by VBM (Fig. 2).

Model 2: digit span was associated with a slightly smaller temporo-parietal cluster (peak coordinate x = -34, y = -38, z = 16, Z-score = 7.70, cluster size = 5,861 voxels). In this model the cluster did not include insular, basal ganglia and frontal regions.

Figure 2. Regions associated with digit span impairments according to VBM and our lesion identification method.

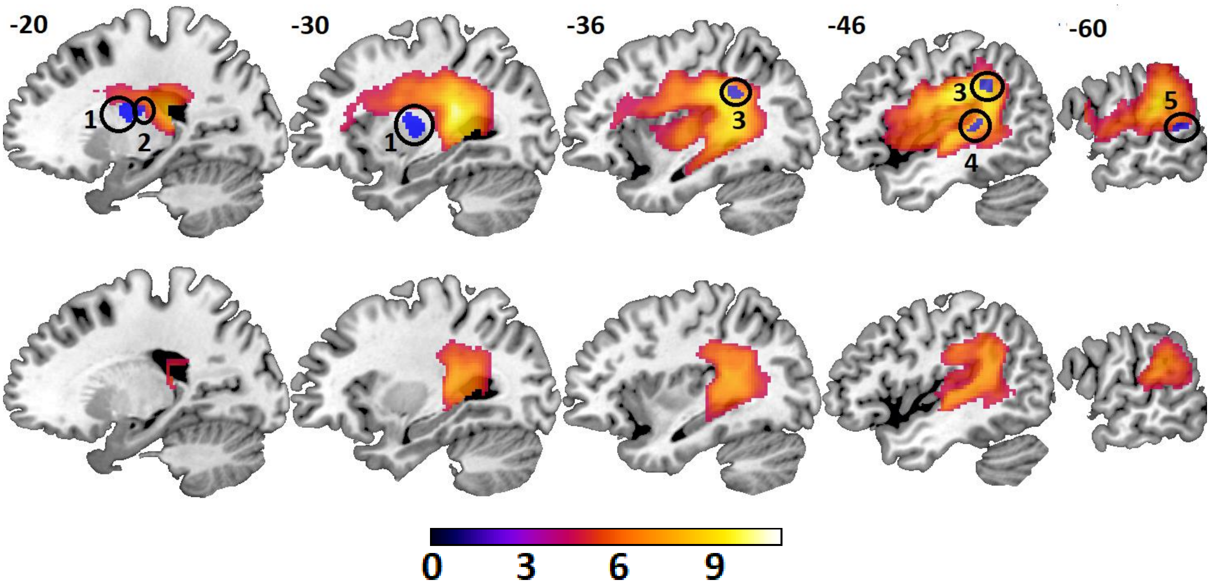

Colour map of areas associated with digit span impairment according to VBM Model 1 (Top) and Model 2 (Bottom), together with the ROIs identified by our lesion identification technique (five regions circled and numbered in black, top panel). The 5 ROIs correspond to those presented in the Results section Fig. 1 (1 = patient PS2002, 2 = patient PS2081, 3 = patient PS1782, 4 = patient PS2095, 5 = patient PS1912).

Sagittal slices are in MNI space. x values are indicated above each slice. Scaled colours representing T-scores are indicated in the bar below. VBM results obtained at $p < 0.05$ FWE-corrected across the entire search volume.

Discussion

These analyses shows that (i) the region identified by VBM was almost as large as the entire left MCA territory, making it extremely nonspecific, and (ii) with the exception of ROI 1 (in Fig. 2) all our ROIs were within the extensive VBM region. Critically, a highly significant relationship between digit span ability and brain structure in multiple voxels does not indicate that all, or even most, voxels are functionally related to digit span ability. Instead, the relationship could be the consequence of damage that co-occurred with that to digit span regions. In contrast, our lesion identification approach focused on the smallest lesions associated with digit span impairment. This (i) minimises the inclusion of brain regions that are not related to digit span but are located in regions that are damaged with digit span regions, and (ii) allows us to identify regions, within the VBM cluster, where damage is sufficient to impair digit span.

References

1. Ashburner J, Friston KJ. Voxel-based morphometry - The methods. *Neuroimage*. 2000;11(6):805-821. doi:10.1006/nimg.2000.0528
2. Tyler LK, Marslen-Wilson W, Stamatakis EA. Dissociating neuro-cognitive component processes: voxel-based correlational methodology. *Neuropsychologia*. 2005;43(5):771-778.
3. Mehta S, Grabowski TJ, Trivedi Y, Damasio H. Evaluation of voxel-based morphometry for focal lesion detection in individuals. *Neuroimage*. 2003. doi:10.1016/S1053-8119(03)00377-X
4. Leff AP, Schofield TM, Crinion JT, et al. The left superior temporal gyrus is a shared substrate for auditory short-term memory and speech comprehension: evidence from 210 patients with stroke. *Brain*. 2009;132(12):3401-3410. doi:10.1093/brain/awp273
5. Fridriksson J, Yourganov G, Bonilha L, Basilakos A, Den Ouden DB, Rorden C. Revealing the dual streams of speech processing. *Proc Natl Acad Sci U S A*. 2016. doi:10.1073/pnas.1614038114
6. Sperber C, Karnath HO. Impact of correction factors in human brain lesion-behavior inference. *Hum Brain Mapp*. 2017. doi:10.1002/hbm.23490
7. Flandin G, Friston KJ. Topological Inference. In: *Brain Mapping: An Encyclopedic Reference*. Vol 1. Elsevier Inc.; 2015:495-500. doi:10.1016/B978-0-12-397025-1.00322-5
